# Enhancing powdery mildew resistance in soybean by targeted mutation of *MLO* genes using the CRISPR/Cas9 system

**DOI:** 10.1186/s12870-023-04549-5

**Published:** 2023-11-03

**Authors:** Thao Phuong Bui, Huy Le, Dong Thi Ta, Cuong Xuan Nguyen, Ngoc Thu Le, Truong Thi Tran, Phuong Van Nguyen, Gary Stacey, Minviluz G. Stacey, Ngoc Bich Pham, Ha Hoang Chu, Phat Tien Do

**Affiliations:** 1 Institute of Biotechnology, Vietnam Academy of Science and Technology, Hanoi, Vietnam; 2 University of Science and Technology of Hanoi, Vietnam Academy of Science and Technology, Hanoi, Vietnam; 3 Present address: Department of Biology, Washington University in St. Louis, St. Louis, USA; 4 Division of Plant Sciences, University of Missouri, Columbia, MO 65211 USA; 5 Legumes Research and Development Center, Vietnam Academy of Agriculture Science, Hanoi, Vietnam; 6 Graduate University of Science and Technology, Vietnam Academy of Science and Technology, Hanoi, Vietnam

**Keywords:** CRISPR/Cas9, *GmMLO* genes, Powdery mildew resistance, Soybean, Targeted mutation

## Abstract

**Background:**

Powdery mildew is a major disease that causes great losses in soybean yield and seed quality. Disease-resistant varieties, which are generated by reducing the impact of susceptibility genes through mutation in host plants, would be an effective approach to protect crops from this disease. The *Mildew Locus O (MLO)* genes are well-known susceptibility genes for powdery mildew in plant. In this study, we utilized the CRISPR/Cas9 system to induce targeted mutations in the soybean *GmMLO* genes to improve powdery mildew resistance.

**Results:**

A dual-sgRNA CRISPR/Cas9 construct was designed and successfully transferred into the Vietnamese soybean cultivar DT26 through *Agrobacterium tumefaciens*-mediated transformation. Various mutant forms of the *GmMLO* genes including biallelic, chimeric and homozygous were found at the T0 generation. The inheritance and segregation of CRISPR/Cas9-induced mutations were confirmed and validated at the T1 and T2 generations. Out of six *GmMLO* genes in the soybean genome, we obtained the *Gmmlo02/Gmmlo19/Gmmlo23* triple and *Gmmlo02/Gmmlo19/Gmmlo20/Gmmlo23* quadruple knockout mutants at the T2 generation. When challenged with *Erysiphe diffusa*, a fungus that causes soybean powdery mildew, all mutant plants showed enhanced resistance to the pathogen, especially the quadruple mutant. The powdery mildew severity in the mutant soybeans was reduced by up to 36.4% compared to wild-type plants. In addition, no pleiotropic effect on soybean growth and development under net-house conditions was observed in the CRISPR/Cas9 mutants.

**Conclusions:**

Our results indicate the involvement of *GmMLO02*, *GmMLO19*, *GmMLO20* and *GmMLO23* genes in powdery mildew susceptibility in soybean. Further research should be conducted to investigate the roles of individual tested genes and the involvement of other *GmMLO* genes in this disease infection mechanism. Importantly, utilizing the CRISPR/Cas9 system successfully created the *Gmmlo* transgene-free homozygous mutant lines with enhanced resistance to powdery mildew, which could be potential materials for soybean breeding programs.

**Supplementary Information:**

The online version contains supplementary material available at 10.1186/s12870-023-04549-5.

## Background

Soybean [*Glycine max* (L.) Merrill] is one of the most important economic legume crops that is grown and consumed all over the world as a source of protein and oil for animal feed and human food [1]. However, global soybean production is seriously threatened by many diseases, one of which is powdery mildew caused by an obligate biotrophic fungus *Erysiphe diffusa* (Cooke & Peck) (syn. *Microsphaera diffusa* (Cooke & Peck) [2–4]. Powdery mildew is considered a major soybean disease [5] that negatively impacts yield in the largest soybean production countries such as the United States, Canada, Brazil, China and Germany. The average, annual yield loss was estimated at 13% because of this disease [6]. However, when environmental conditions are optimal for fungal growth, the yield loss of some susceptible varieties can approach 35%-60% [7, 8].

The use of resistant varieties would be an effective method to protect plant from powdery mildew. Although resistant genes (R-genes) against powdery mildew have been identified in some plant species. Up to now, only one R-gene named the *resistance-to-M. diffusa 1* (*Rmd1*) was cloned and characterized in soybean [9], however, it is still a challenge to introduce the R-gene to the local cultivar. Reducing the impact of susceptibility genes (S-genes) through mutation in host plants is now considered as an effective alternative approach for disease resistant breeding. Recessive mutations of S-genes can limit pathogen infection of host plants and provide durable and broad-spectrum resistance [10]. The *Mildew Locus O (MLO)* genes are well-known S-genes for powdery mildew in different plant species. The *MLO* genes encode seven-transmembrane domain proteins [11], which are conserved throughout monocots and dicots [12]. These proteins are located in the plasma membrane and contain a 20 amino acid-long calmodulin (CaM)-binding domain, which is required for susceptibility to powdery mildew infection [13, 14]. Previous reports demonstrated the important role of *MLO* genes in the susceptibility to powdery mildew in barley [15, 16], *Arabidopsis* [17], tomato [18], pea [19, 20], pepper [21], bread wheat [22, 23], rose [24], apple [25], and grapevine [26, 27]. In addition, loss-of-function mutant alleles of *MLO* genes generated by different mutagenic approaches, such as chemical, RNAi, TALLEN and TILLING provided complete or enhanced resistance to powdery mildew in various plant species [17, 22, 28]. Therefore, inducing loss-of-function mutations in *MLO* is a potential strategy to improve powdery mildew resistance in important crops.

Recently, CRISPR/Cas9 was mentioned as the most effective and precise approach for trait improvement in crop plants [29]. This system has been successfully utilized to induce targeted mutations of *MLO* genes for enhancing powdery mildew resistance in wheat, grapevine, and tomato [27, 30–32]. In soybean, 39 *GmMLO* genes were previously predicted using comparative phylogenetic analysis from soybean and *Arabidopsis* genomes but their respective functions have not been defined [33, 34]. The aims of this study were to utilize the CRISPR/Cas9 system for inducing targeted mutations of selected *GmMLO* genes in a Vietnamese elite soybean cultivar DT26, and investigate their functions in powdery mildew susceptibility. The CRISPR/Cas9-induced *Gmmlo* mutant lines were generated and the inheritance of *GmMLO* mutations was assessed through generations. The homozygous mutant lines were subsequently identified for powdery mildew challenges. Moreover, pleiotropic effects of *GmMLO* mutations on soybean growth and development were analyzed under the net-house condition. The results here would provide a potential system to generate local soybean cultivars with enhanced powdery mildew resistance.

## Results

### Target selection and CRISPR/Cas9 vector validation

*MLO* which encodes a membrane-associated protein with seven transmembrane domains is conserved throughout monocots and dicots. Loss-of-function *mlo* mutations confer durable and broad-spectrum resistance to powdery mildew in various crop species [12, 28]. In soybean, there are 39 *MLO* genes, of which *GmMLO2, GmMLO10, GmMLO18, GmMLO19, GmMLO20,* and *GmMLO23* are closely related to *AtMLO2, AtMLO6* and *AtMLO12*, which were shown to play a role in powdery mildew susceptibility in *Arabidopsis thaliana* [34]. Publicly-available soybean RNA-Seq Atlas indicated that the above mentioned *GmMLO* genes are expressed in various soybean tissues at low levels (Fig. S1). To investigate the roles of these soybean *MLO* genes in powdery mildew resistance, we employed the CRISPR/Cas9 system to induce knock-out mutations in all six *GmMLO* genes.

**Fig. 1:**
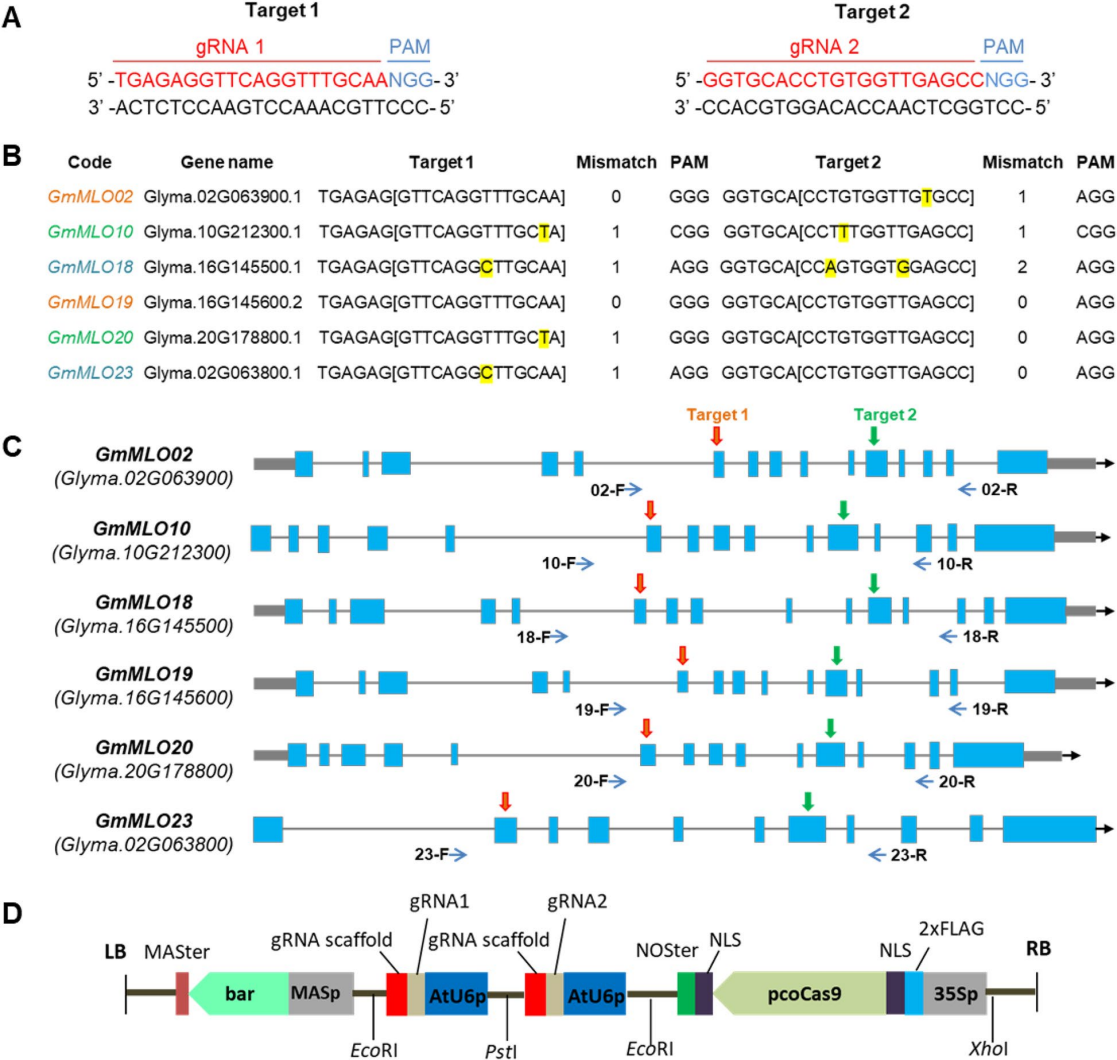
*GmMLO* gene maps, gRNA sequences, target locations and CRISPR/Cas9 vector. **A** gRNA and PAM sequences. **B** *GmMLO* genes and target sequences. Letters in yellow indicate mismatches that are within the gRNA core (PAM-1 to PAM -14). Different colors in the code column indicate different pairs of homologous genes. **C** *GmMLO* gene structures and target locations, primers for genotyping are indicated by arrows. **D** T-DNA region for soybean transformation. *bar*, herbicide resistant gene as selection marker; *pcoCas9*, *Cas9* codon-optimized gene, driven by 35SPPDK promoter (35Sp) and two sgRNAs driven by *Arabidopsis U6* promoter *(*AtU6p*)*; MASp, *Manopine Synthase* promoter; MASter, *Manopine Synthase* terminator; NOSter, *Nopaline synthase* terminator; LB/RB, Left and Right Border

Based on the Williams 82 (W82) reference genome, we identified two potential target sites which were highly conserved and located within the exons of these six *MLO* genes (Fig. 1A-C). The sgRNA target 1 contained a single-mismatch in the *GmMLO20*, *GmMLO23, GmMLO18, GmMLO10* while the sgRNA target 2 contained a single-mismatch in the *GmMLO02, GmMLO10* and double-mismatch in the *GmMLO18* (Fig. 1B). Sequencing results of these *MLO* genes in the Vietnamese elite soybean (DT26) showed that the target sites in *GmMLO02, GmMLO18*, *GmMLO19*, *GmMLO20*, *GmMLO23* were identical to the reference Williams 82 genome. Whereas a single nucleotide polymorphism was found in the sgRNA target 1 of *GmMLO10*. Despite these mismatches, these were the most conserved target sites available for all six genes; thus, we selected these two target sites to generate a dual-sgRNA CRISPR/Cas9 vector reagent (Fig. 1D).

To validate the efficacy of the dual-sgRNA-CRISPR/Cas9 construct, the CRISPR/Cas9 vector was mobilized into *Agrobacterium rhizogenes* K599 strain for soybean hairy root transformation. Ten independent in vitro hairy root lines were used for mutagenesis analysis by PCR- agarose gel electrophoresis. Low mobility DNA bands were observed in 30% of the root samples indicating that large deletion(s) was induced in *GmMLO20* (Fig. S2A). Sequencing of PCR amplicons derived from one hairy root line (HR1) indeed showed a 1214-bp deletion in *GmMLO20* (Fig. S2B). Therefore, these results indicated that the CRISPR/Cas9 construct was sufficiently efficent for stable soybean transformation.

### Generation of transgenic soybean and characterization of *GmMLO* induced mutations

**Fig. 2:**
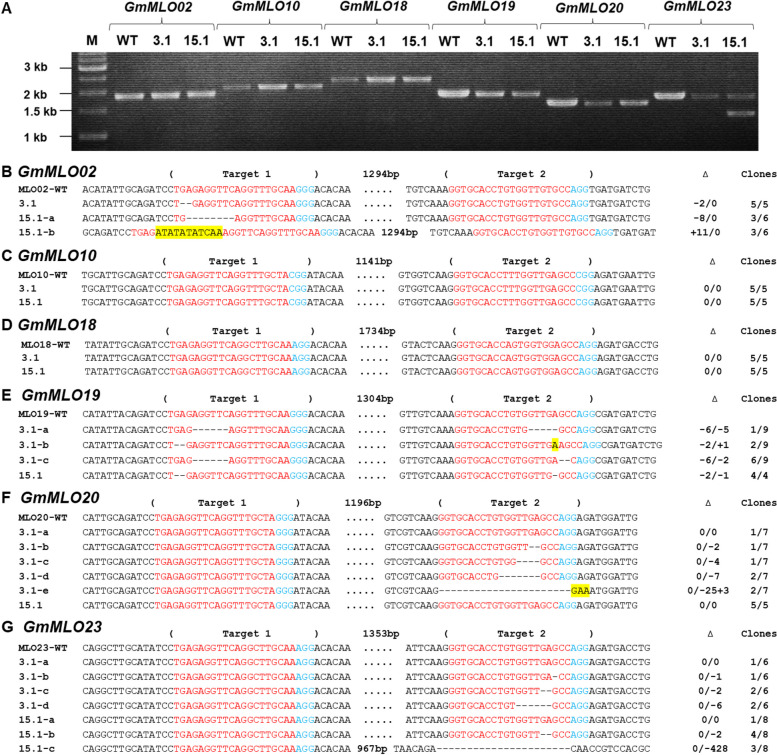
Identification and characterization of induced *MLO* mutations in T0 transgenic soybean plants. **A** Gel electrophoresis of PCR-amplicons of *GmMLO* target expanding regions. WT, non-transgenic wild-type plant; 3.1, 15.1, transgenic lines; M, 1 kb DNA ladder. **B**-**G** Sequence alignment of targeted regions in *GmMLO* genes (*GmMLO02*, *GmMLO10*, *GmMLO18*, *GmMLO19*, *GmMLO20* and *GmMLO23*) of T0 transgenic lines. Target sequences and PAMs are indicated in red and blue color, respectively. Inserted nucleotides are shown in yellow. a/b/c/d/e indicates different alleles for each T0 line; ∆ indicates targeted sequence changes: 0 for no change, -for deletion, + for insertion. Clones indicate number of colonies with the respective alleles out of total of clones sequenced

Two transgenic lines 3.1 and 15.1 were generated using *Agrobacterium*-mediated transformation. The presence of transgenes was confirmed by herbicide leaf painting (Fig. S3F, G) and PCR amplification using primers for the *bar* gene and the 35S promoter spanning sequences (Table S3). CRISPR/Cas-induced mutations in the six *GmMLO* genes were first evaluated by PCR using gene specific primers followed by agarose gel electrophoresis (Fig. 2A). However, low mobility PCR amplicons, indicative of large deletions, were found only for the *GmMLO23* gene in the 15.1 line. Sanger sequencing of wild-type and low mobility amplicons indicated biallelic mutations of -428 bp and -2 bp alleles in the *GmMLO23* gene of the 15.1 line. We further used Sanger sequencing of PCR amplicons with wild-type mobility to determine if small deletions were induced that were undetectable by gel electrophoresis. Indeed, various small insertions (from + 1 to + 11 bp) and deletions (from -1 bp to -25 bp) were observed at the target sites of the *GmMLO* genes in the two transgenic lines (Fig. 2B-G). We also observed chimeric mutations (more than two different alleles) in certain *GmMLO* genes, which suggests that CRISRP/Cas9 activity may occur late during shoot development. However, we found no mutation in the *GmMLO10* and *GmMLO18* genes of both two transgenic lines. In summary, the 3.1 line carried a homozygous mutation of the *GmMLO02* gene and chimeric forms for the other three tested *GmMLO* genes including *GmMLO19, GmMLO20* and *GmMLO23*. For the 15.1 line, homozygous, biallelic and chimeric mutant forms were found in the *GmMLO19, GmMLO02* and *GmMLO23* genes, respectively, but no indel was observed in the *GmMLO20* gene (Table S2).

### Assessment of the inheritance of *GmMLO* mutations

**Fig. 3:**
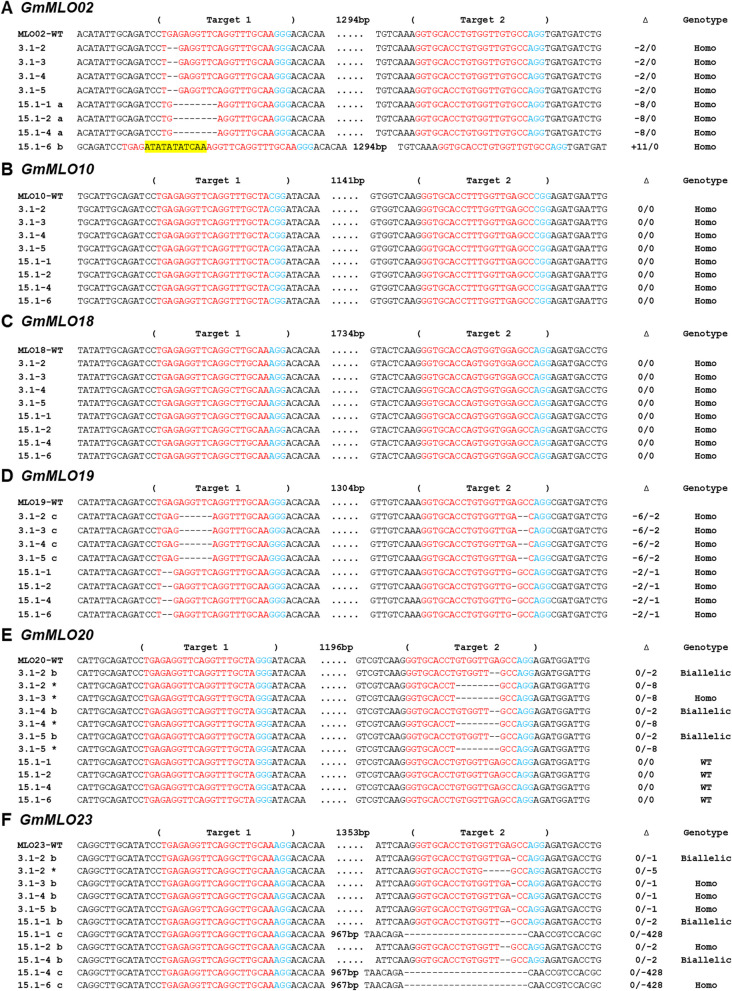
Inheritance and segregation of targeted induced mutations at T1 generation. Target sequences and PAMs are indicated in red and blue color, respectively. Inserted nucleotides are shown in yellow. a/b/c indicates different alleles inherited from T0 for each T1 line; * indicates new alleles appeared at T1 generation; ∆ indicates targeted sequence changes: 0 for no change,—for deletion, + for insertion

Gel electrophoresis and sequencing were conducted to assess the inheritance of CRISPR/Cas9-induced mutations at the T1 generation (Figs. S4, 3A-F). The large indel (-428 bp) in the *GmMLO23* gene was passed to T1 progeny of the 15.1 line and visually observed by DNA band shifts on the agarose gel (Fig. S4). The inheritance of certain induced mutations including -2/0 (for line 3.1), -8/0 and + 11/0 (for line 15.1) of *GmMLO02;* -6/2 (for line 3.1) and -2/1 (for line 15.1) of *GmMLO19;* 0/-2 (for line 3.1) of *GmMLO20;* and 0/-1 (for line 3.1), 0/-2 and 0/-428 (for line 15.1) of *GmMLO23* genes was confirmed by Sanger sequencing (Fig. 3)*.* The absence of other indels indicated that they were chimeric mutations at the T0 generation that did not pass through the germline. No mutation was found in the *GmMLO10* and *GmMLO18* genes at the T1 generation (Fig. 3C, D). In addition, we also detected new indels in the T1 plants suggesting that late CRISPR/Cas activity occurring in the T0 plants that were subsequently passed through the germline (Fig. 3E, F). Particularly, 0/-8 allele was observed in the *GmMLO20* gene of the 3.1 offspring, while 0/-5 mutations occurred in the *GmMLO23* gene from some T1 plants of this T0 line.

Four T1 plants (3.1–3, 3.1–5, 15.1–2 and 15.1–6) harboring homozygous mutations in *GmMLO* genes were used for offspring analysis. Of these, 3.1–3 carried homozygous mutations in four *GmMLO* genes (*GmMLO02, GmMLO19*, *GmMLO20* and *GmMLO23*), while 3.1–5 contained biallelic mutations in *GmMLO20* and homozygous mutant alleles in three *GmMLO* genes (*GmMLO02, GmMLO19* and *GmMLO23*) (Fig. 3). Meanwhile, both 15.1–2 and 15.1–6 were homozygous for induced mutations in *GmMLO02, GmMLO19* and *GmMLO23* genes, but carried no mutation in the *GmMLO10*, *GmMLO18* and *GmMLO20* genes*.* Sequencing data of selected T2 plants showed all CRIRSPR/Cas9-induced mutations from the four T1 lines, which demonstrated stable inheritance of these indels (Fig. S5). Progenies derived from four (4) T2 lines (3.1–3-41, 3.1–5-44, 15.1–2-2, and 15.1–6-4), representing various combinations of *GmMLO* mutations, were selected for fungal challenges (Table 1).

**Table 1:** Mutant characterization of T2 CRISPR/Cas9-edited soybeans

Events	Induced indels at Target 1/Target 2					
	*GmMLO02*	*GmMLO10*	*GmMLO18*	*GmMLO19*	*GmMLO20*	*GmMLO23*
3.1–3-41	-2/0	WT	WT	-6/-2	0/-8	0/-1
3.1–5-44	-2/0	WT	WT	-6/-2	0/-2	0/-1
15.1–2-2	-8/0	WT	WT	-2/-1	WT	0/-2
15.1–6-4	+ 11/0	WT	WT	-2/-1	WT	0/-248

### Evaluation of powdery mildew resistance of *GmMLO* mutant soybeans

**Fig. 4:**
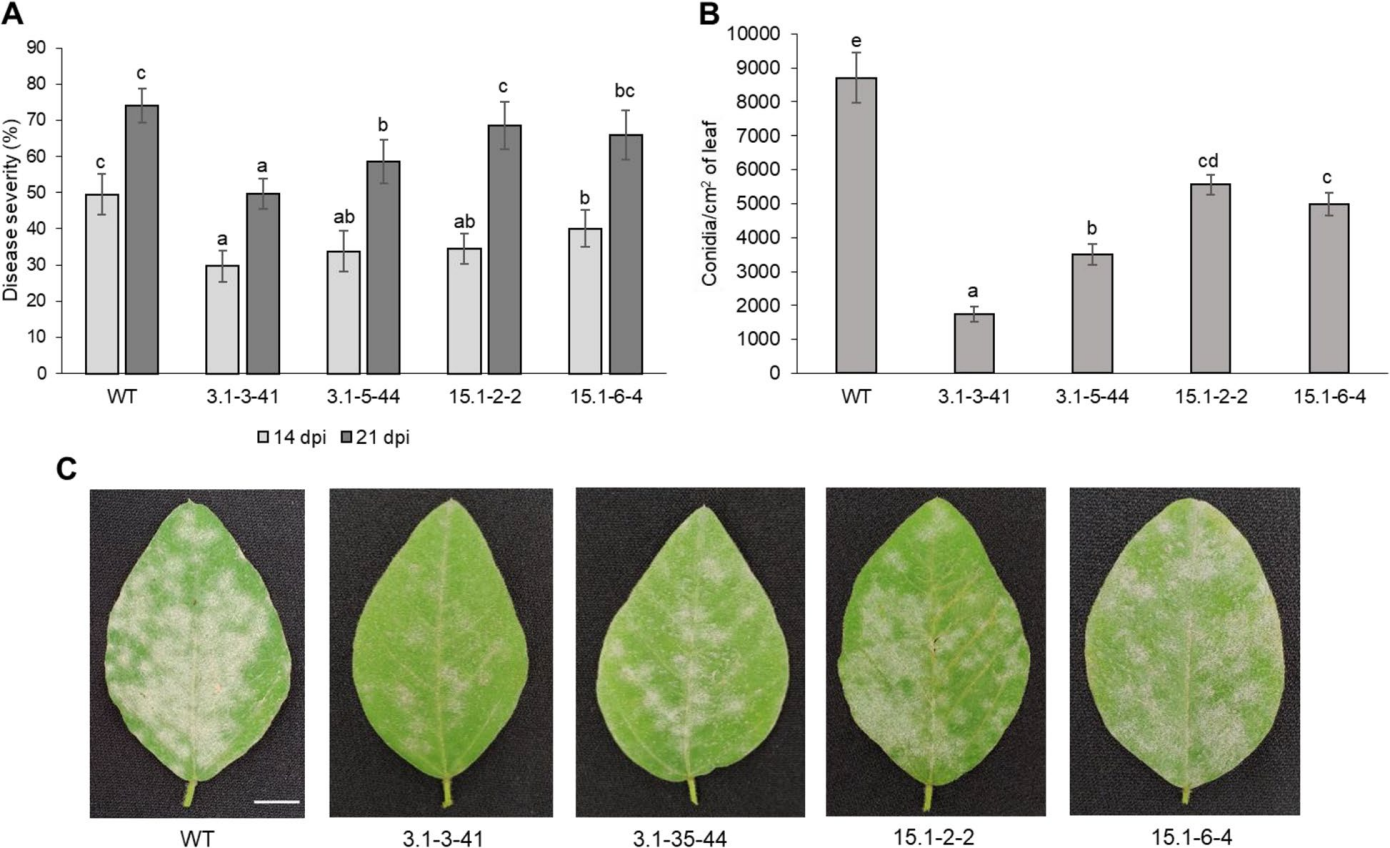
Soybean powdery mildew resistant assessment. **A** Disease severity assessment at 14 dpi and 21 dpi. Error bars indicate standard deviations, *n* = 5–6. Each time point was analyzed independently using one-way ANOVA followed by a post hoc Turkey’s test (*P* < 0.05). **B** Quantification of conidia per cm^2^ leaf surface at 21 dpi. Error bars indicate standard deviations, *n* = 6. Statistical analysis was done using one-way ANOVA followed by a post hoc Turkey’s test. Significant difference was considered at *P* < 0.05. **C** The representative leaves were collected 21 days after inoculation. Scale bar = 1 cm. WT, control line (DT26 cultivar); 3.1–3-41, 3.1–5-44, 15.1–2-2, 15.1–6-4, T2 offspring *Gmmlo* soybean mutant lines

*E. diffusa* conidia from powdery mildew infected soybean leaves was isolated and confirmed by morphological characterization and 16S sequence analysis (Fig. S6), then used for the artificial infection of wild-type and T3 mutant plants. At 14 days post inoculation (dpi), all mutant plants showed reduced disease severity (19.1% to 40% reduction) compared to wild-type control plants (Fig. 4A; Table 2), with plants derived from 3.1–3-41 line showing the highest resistance to *E. diffusa* infection. At 21 dpi, a significant decrease in disease severity was only observed in 3.1–3-41 and 3.1–5-44 plants. In addition to decreased disease severity, the conidial density in the infected leaf surfaces was dramatically reduced in all mutant soybean lines (1.5 to 5 times), which indicated the inhibition of fungal development (Fig. 4B, C).

**Table 2:** Powdery mildew symptom reduction of mutant lines compared to WT

Lines	Disease reduction (%)		Average reduction (%)
	14 dpi	21 dpi	
3.1–3-41	40.0	32.8	36.4
3.1–5-44	31.7	20.8	26.3
15.1–2-2	30.5	7.5	19.0
15.1–6-4	19.1	10.9	15.0

Note: Disease reduction was calculated as WT disease severity subtracted mutant disease severity divided by WT disease severity and × 100. 3.1–3-41, 3.1–5-44, 15.1–2-2, 15.1–6-4, T2 offspring *Gmmlo* soybean mutant lines

**Fig. 5:**
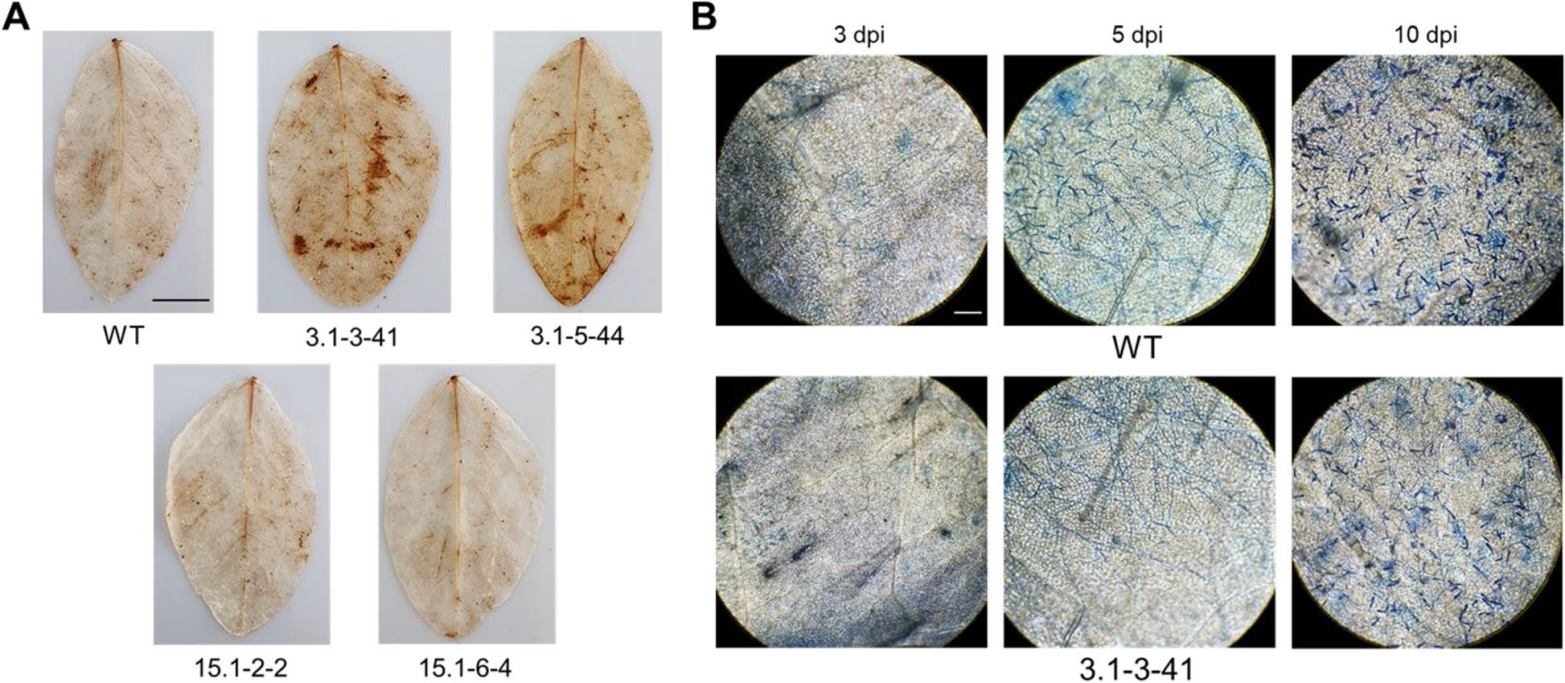
Powdery mildew fungal development and damages to soybean leaves. **A** The accumulation of hydrogen peroxide on powdery mildew infected leaves at 24 hpi using DAB staining method. WT, control line (DT26 cultivar); 3.1–3-41, 3.1–5-44, 15.1–2-2, 15.1–6-4, T2 offspring *Gmmlo* soybean mutant lines. Scale bar = 1 cm. **B** *E. diffusa* hyphae development and conidia formation in the wild-type leaves and in the T2 offspring targeted mutant line 3.1–3-41 at 3, 5 and 10 dpi. Scale bar = 100 µm

3, 3'-diaminobenzidine (DAB) staining showed stronger H_2_O_2_ accumulation with more brown spots in infected leaves of lines 3.1–3-41 and 3.1–5-44 as compared to wild-type plants, indicating a stronger reactive oxygen species (ROS) response to fungal infection. In contrast, there was no visible difference in DAB staining between the infected leaves of two lines 15.1–2-2, 15.1–6-4 and wild-type leaves (Fig. 5A). Histological analysis of the 3.1–3-41 line, which showed the lowest infection levels of soybean powdery mildew, also exhibited a delay of *E. diffusa* hyphae development (Fig. 5B). Particularly, at 3 dpi, hyphae were found on the infected leaf surfaces of wild-type plants, but not in the 3.1–3-41 line. At 5 dpi, both developed hyphae and conidiophores were observed on the leaf surfaces of wild-type plants, while only hyphae were found on the leaves of the 3.1–3-41 line. At 10 dpi, the conidial density was much higher in wild-type leaves as compared to the 3.1–3-41 line. We further assessed the enhanced powdery mildew resistance of the mutant soybean lines under net-house farming conditions with high pathogenic pressure of *E. diffusa*. The disease severity was measured and recorded at 2.6 and 3.5 for the 3.1–3-41 and 3.1–5-44 lines, respectively. However, severity increased up to 4.7 in infected plants from the 15.1–2-2 and 15.1–6-4 mutant lines, as well as the wild-type (Fig. S7). Altogether, our results indicated that the two mutant lines 3.1–3-41 and 3.1–5-44, which carry homozygous mutations of *GmMLO02, GmMLO19, GmMLO20* and *GmMLO23* genes, showed the least susceptibility to powdery mildew infection.

### Growth and development of soybean homozygous mutant lines

**Fig. 6:**
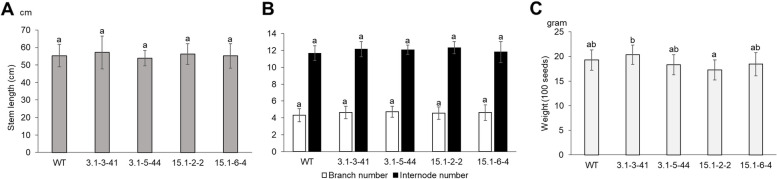
Growth and development of T2 offspring *Gmmlo* mutant soybeans under the net-house conditions. **A** Stem length. **B** Branch and internode number. **C** Weight of 100 seeds. WT, control line (DT26 cultivar); 3.1–3-41, 3.1–5-44, 15.1–2-2, 15.1–6-4, T2 offspring *Gmmlo* soybean mutant lines. Error bars indicate standard deviations, *n* = 9–14. Statistical analysis was done using one-way ANOVA followed by a post hoc Turkey’s test. Significant difference was considered at *P* < 0.05

The morphology and agronomic traits of T3 plants carrying homozygous mutations of *GmMLO02, GmMLO19, GmMLO20* and *GmMLO23* were assessed under net-house conditions (Fig. 6). No significant differences in plant height, branch and internode number were observed between mutants and wild-type plants (Fig. 6A, B). For agronomical traits, the seed weights varied slightly between the wild-type and the mutant lines, but we found no statistically significant difference (Fig. 6C). In addition, mutant soybean plants showed no change in total pods per plant as compared to the wild-type (around 30 pods per plant), except line 15.1–6-4, which had about 22 pods per plant (Fig. S8A). However, this mutant line also exhibited the highest rate of 3-seeded pods (Fig. S8B). Altogether, targeted mutations in *GmMLO* genes had no obvious negative effects on soybean growth and development under net-house conditions.

### Identification of transgene-free homozygous mutants

Transgene-free mutant soybeans were screened at the T1 and T2 generations using herbicide leaf-painting (Fig. S9A) and PCR with specific primers for *bar* gene and for transgene region spanning the pFGC vector and 35SPPDK promoter of pco*Cas9* (Fig. S9B; Table S1). All tested T1 plants were resistant to herbicide. In addition, PCR results also confirmed the presence of transgenes in these plants (Fig. S9B; Table S3). At the T2 generation, we identified 4 plants from the T0 line 3.1 were negative for the presence of transgenes and susceptible to herbicide (Fig. S9B; Table S3). Of which, two lines 3.1–3-41 and 3.1–5-44 were then confirmed to carry quadruple null mutations in four *GmMLO* genes. In line with previous reports, our results indicate that transgene-free mutant soybean could be obtained as early as the T2 generation using the CRISPR/Cas9 system.

## Discussion

Inducing loss-of-function mutations in *MLO* genes using the CRISPR/Cas9 system is a promising approach to generate powdery mildew resistant cultivars in important crops, such as wheat [22], grapevine [27, 35], and tomato [30, 31]. In this study, we utilized the dual-gRNA CRISPR/Cas9 system to simultaneously knock-out the four soybean *MLO* homologs—*GmMLO02, GmMLO19, GmMLO20,* and *GmMLO23—*in the Vietnamese soybean elite cultivar DT26, and resulted in elevated resistance to powdery mildew.

In soybean, six out of 39 putative *GmMLO* genes—*GmMLO02, GmMLO10, GmMLO18, GmMLO19, GmMLO20,* and *GmMLO23* were found to be orthologous to three *AtMLO* genes (*AtMLO*2, *AtMLO*6, *AtMLO*12), which are required for resistance to powdery mildew in *Arabidopsis* [17]. Thus, we designed a plasmid vector for simultaneous targeting of all six *GmMLO* genes. The use of a dual-gRNA CRISPR/Cas9 system increases editing frequency either single or multiple genes in soybean [36–38]. We found the dual-gRNA CRISPR/Cas9 system induced mutations in *GmMLO02*, *GmMLO19, GmMLO20* and *GmMLO23* in the two soybean events, however, no mutation was found in the *GmMLO10* and *GmMLO18* in these events (Fig. 2; Table S2). The lack of mutations in these latter two genes may be due to mismatches in the ‘seed’ sequence (10–12 bp proximal to the PAM), which could significantly reduce Cas9's ability to bind and cleave its target [39]. Indeed, we found one to two mismatches located in the seed region of the targets of the *GmMLO10* and *GmMLO18* in the soybean cultivar DT26 (Fig. 1B). In addition, we also found a mismatch in target 1 of *GmMLO20* and *GmMLO23* or in target 2 of *GmMLO02* inhibited the cleavage activity of Cas9 in soybean plants.

Functional *MLO* genes associated with powdery mildew resistance are exclusively grouped in clade V for dicots [12]. In this study, we showed that at least four members of *GmMLO* in this clade are involved in powdery mildew resistance in soybean. We found that not only the quadruple mutants (*Gmmlo02/Gmmlo19/Gmmlo20/Gmmlo23*) but also the triple mutant (*Gmmlo02/Gmmlo19/Gmmlo23*) exhibited significantly enhanced *E. diffusa* resistance compared to the wild-type at 14 dpi (Fig. 4A). However, the quadruple mutants showed more resistant to the pathogen compared to the wild-type and the triple mutants at 21dpi, as exemplified by fewer conidia formation and hyphae development of *E. diffusa* (Figs. 4B, C, and 5B). In addition, H_2_O_2_ production and accumulation, visualized as brownish precipitates upon DAB staining, were more prominent in mildew-infected leaf of the quadruple *GmMLO mutant* lines at 21 hpi (Fig. 5A). The correlation between the production of H_2_O_2_ and resistance to powdery mildew in our soybean mutants is similar to reports in barley [40], cucumber [41] and grapevine [27] where H_2_O_2_ accumulation, and subsequent host cell death, is one of defense mechanisms in *mlo* plants to powdery mildew [17, 28, 40]. Hence, our results showed these four *GmMLO* genes are functionally conserved and contribute to powdery mildew resistance in soybean. Functional redundancy, especially unequal genetic redundancy, of *MLO* members in contribution to powdery mildew resistance was reported for *Arabidopsis*, grape and tomato [17, 26, 42]. In *Arabidopsis*, *Atmlo2* single mutant plants displayed partial powdery mildew resistance, whereas *Atmlo2/Atmlo6/Atmlo12* triple mutant plants were fully resistant. A similar scenario was observed in tomato, where the *SlMLO1* is the major powdery mildew susceptibility factor, and *SlMLO5* and *SlMLO8*, have minor function [42]. In grape, knock-down of at least three *MLO* genes including *VvMLO7*, *VvMLO11* and *VvMLO6* significantly reduced powdery mildew severity [26]. Taken together, our data suggest that *GmMLO02*, *GmMLO19*, and *GmMLO23* are functionally redundant with *GmMLO20* as powdery mildew susceptibility factors in soybean. Complete resistance to powdery mildew was obtained in the knock-out and knock-down of three *MLO* genes in *Arabidopsis* and tomato, respectively [17, 42]. However, our mutant plants did not exhibit complete resistance to powdery mildew. This may indicate that additional *GmMLO* genes, other than the four mutated in our study, also function to some extent in the susceptibility of soybean to powdery mildew infection. Further researches need to be performed to clarify the contribution of single tested *GmMOL* genes as well as the interaction of these genes in the mechanism of powdery mildew resistance in soybean.

In plants, *MLO* genes have been implicated in various physiological processes [12]. Disruption of these genes were accompanied by undesired pleiotropic effects such as leaf chlorosis and reduced grain yield in barley and wheat [14, 21, 31], reduced growth in *A. thaliana* [17], smaller plant size in pepper [21], as well as senescence-like chlorosis and necrosis in grapevine [27]. In our study, no pleiotropic phenotype was observed in the *Gmmlo* mutants under net-house conditions. The resistant lines carrying loss-of-function mutations in four *GmMLO* genes, i.e., *GmMLO02*, *GmMLO19*, *GmMLO20* and *GmMLO23,* showed no obvious difference in morphology, development and seed production compared to wild-type plants. The transgene-free *Gmmlo* mutant soybean lines with the highest resistance to powdery mildew created in this study should be useful genetic materials for breeding programs for increased disease resistance.

## Conclusions

In summary, we were successful in using a dual-gRNA CRISPR/Cas9 system to simultaneously knock-out the four soybean *MLO* homologs—*GmMLO02, GmMLO19, GmMLO20,* and *GmMLO23*—in the Vietnamese soybean elite cultivar DT26. The CRISPR/Cas9-induced *Gmmlo* mutant lines exhibited enhanced resistance to soybean powdery mildew. Moreover, the *Gmmlo* transgene-free mutant lines showed no obvious difference in morphology, development and productivity compared to wild-type plants. Our results indicate the involvement of four *GmMLO* genes in soybean powdery mildew susceptibility and provide a potential strategy for improving disease resistance of local soybean cultivars.

## Methods

### CRISPR/Cas9 vector construction

One pFGC-CRISPR/Cas9 vector carrying dual sgRNAs, each independently driven by a *Arabidopsis thaliana* AtU6 promoter, was constructed for simultaneously inducing targeted mutations of multiple selected *GmMLO* genes in soybean. Particularly, AtU6 promoter and gRNA scaffold were derived from the pBlu/gRNA vector, a gift from Robert Stupar's laboratory (RRID: Addgene_59188). Double-stranded DNA oligonucleotides of sgRNA were cloned into the pBlu/gRNA vector by *Bpi*I sites. The expected fragments (AtU6-gRNA- scaffold) were excised by *EcoR*I sites and used for gel purification. The cassette of a plant-codon-optimized Cas9 driven by 35S promoter was generated from the HBT-pcoCas9 vector, a gift from Jen Sheen's laboratory (RRID: Addgene_52254) using *EcoR*I and *Xho*I sites*.* All designed cassettes were assembled in the pFGC5941 backbone by *EcoR*I sites to generate the final construct pFGC5941-gRNA1-gRNA2-Cas9. The designed construct was validated and confirmed by Sanger sequencing.

### Soybean hairy root transformation

Soybean in vitro hairy root transformation was performed following a previously reported method [43] to evaluate the targeted editing activity of the designed CRISPR/Cas9 vector. Briefly, cotyledons from 4-day-old seedlings of Vietnamese elite cultivar DT26 obtained from Legumes Research and Development Center, Field Crops Research Institute, Vietnam Academy of Agricultural Sciences were used as explants for infection with* A. rhizogenes* K599 strain harboring the pFGC5941-gRNA1-gRNA2-Cas9 construct. Seven days after co-cultivation, induced soybean hairy roots were transferred to selection medium (MS medium with 3 mg/L glufosinate). Genomic DNA was extracted from herbicide-resistant hairy roots using the CTAB method [44] and used for induced mutant identification and characterization.

### Stable soybean transformation and transgene confirmation

Soybean transformation was performed using *Agrobacterium tumefaciens* via cotyledon node infection as previously described [37, 45] (Fig. S3A-E). Regenerated plants on selection medium were transferred to perlite and vermiculite mixture (1:3 v/v) for acclimatization, then cultured in plastic pots containing TRiBAT® compost (Green Saigon Biotechnology Limited Company, Vietnam) under greenhouse conditions. Glufosinate solution (200 mg/L) was painted onto three trifoliate leaves of each plant for herbicide resistant tests. Genomic DNA of herbicide-resistant plants was extracted by the CTAB method [44] and used for transgene confirmation with specific primers (Table S2).

### Induced mutant identification and characterization

The target spanning regions on the *GmMLO* genes were amplified using specific primers (Table S2) and analysed by 1% agarose gel electrophoresis to detect DNA band shifts. The PCR amplicons were then purified and ligated to the pJET1.2 cloning vector (Thermo Fisher Scientific, USA) for Sanger sequencing by ABI PRISM® 3100 Avant Genetic Analyzer system (Applied Biosystems, USA). The sequencing data were analysed by the FinchTV chromatogram viewer program (Geospiza) and MEGA X [46].

### Plant cultivation and morphological characterization

Mature soybean seeds were imbibed on moist paper for 48 h at 26^o^C, then sown in plant pots (26 cm depth, 21 cm diameter top) containing the TRiBAT® compost mixture (Green Saigon Biotechnology Limited Company, Vietnam) and organic soil (Minh Hiep Thanh Cooperative, Vietnam) (1:3 v/v). Soybean plants were grown under net-house conditions and fertilized with NPK (15:5:20) at the V3 stage, NPK (16:16:16) at 40 and 65 days-old stages. Plant morphological parameters and soybean yield traits including stem length, branch and internode number, total pods, seeds per pod and seed weight were collected and analysed at the R8 stage. Seeds were harvested, dried and stored in seed room at 40% humidity and 4°C for further experiments.

### *E. diffusa* susceptibility assessment

Soybean leaves with symptoms of powdery mildew infection were provided by Legumes Research and Development Center, Field Crops Research Institute, Vietnam Academy of Agricultural Sciences. Conidiophore and conidia of powdery mildew fungus were visually confirmed and isolated from the infected leaves. Fungal genomic DNA was extracted using a EZ-10 Spin Colum Fungal Genomic DNA Mini-Preps Kit (Bio Basic, Canada) and used for species confirmation by PCR with ITS1/PM6 specific primers [47, 48]. The *E. diffusa* isolate was subsequently maintained and propagated on a susceptible soybean under growth chamber condition. The fungus was infected into selected T3 *Gmmlo* mutant plants (n ≥ 5 for each mutant line) at the V2 stage using the leaf brushing method as described by Kang and Mian [49]. Infected plants were kept in a growth chamber (22°C ± 1°C and 100% RH) for 12 h to promote fungal germination, penetration, and development. The disease symptom and severity were recorded and analysed at 14- and 21-days post inoculation (dpi) as previous description by Pessina et al. [25].

For the net-house test, mature seeds of selected T3 mutant lines were directly sown beside the susceptible cultivar showing powdery mildew symptoms. The disease severity was observed and scored at V3, V5 and V7 stages based on the symptom scales (0 to 5 grades) proposed by Tran et al. [50].

### Histological analysis

*E. diffusa* infected leaves were collected at 3, 5 and 10 dpi and submerged in ethanol-acetic acid solution (3:1 v/v) to remove chlorophyll [25]. The treated leaves were stained with 250 µg/mL trypan blue in lactoglycerol solution [lactic acid:glycerol:water 1:1:1 (v/v/v)] for 15 min, then rinsed in the same solution at room temperature as described by Vogel and Somerville [51]. The samples were then mounted and captured under 100X magnification for visualization of hyphae development and conidia germination.

DAB staining was conducted to assess hydrogen peroxide (H_2_O_2_) accumulation in infected leaves at 24 hpi followed the method by Yu et al. [41]. The stained leaves were boiled in ethanol-lactic acid-glycerol (3:1:1 v/v/v) for 20 min and then transferred to pre-chilled 95% ethanol before being photographed.

### Data analysis

Agronomic traits and disease severity data were analyzed with SPSS Statistics software (version 20.0, IBM, Armonk, NY). The mean values and standard deviation of the mean (SD) were calculated and presented reflecting three replicates. Statistical significance was conducted using one-way ANOVA followed by a *post-hoc* Turkey’s test at *P* < 0.05.

### Supplementary Information

**Additional file 1: Fig. S1.** Transcriptomics analysis of *GmMLO *genes in different tissues of soybean plant. Data were obtained from RNA-Seq Atlas of *Glycine max. ***Fig. S2.** Induced mutation analysis of hairy roots. A Gel electrophoresis (agarose 1%) of *GmMLO20 *edited region in wild-type (WT) and hairy root samples (HR1, HR2, HR3) with large deletions. M: 1 kb DNA marker. Shifted bands in lines HR1, HR2 and HR3 indicated the induced mutations of targeted genes. B Sequencing result of the HR1 line for the edited regions of *GmMLO20 *compared to wild-type allele. Target sequences and PAMs are indicated in red and blue, respectively. Δ indicates targeted sequence changes: - for deletion. Clones indicate number of colonies with the respective alleles out of total of clones sequenced. **Fig. S3.** Soybean transformation procedure. A Cotyledons at 5 days on the co-cultivation medium. B, C Shoot induction at 14 and 28 days on the selection medium. D Shoot elongation. E Rooted plants on the rooting medium. F, G Leaf painting using 200 mg/L glufosinate. **Fig. S4.** Gel electrophoresis of PCR-amplicons of *GmMLO *target expanding regions at T1 generation. WT: Non transgenic wild-type plant; 3.1-2 to 3.1-5: T1 plants from 3.1 line; 15.1-1 to 15.1-6: T1 plants from 15.1 line; M: 1 kb DNA ladder. PCR products amplified by specific primers for extended regions of *GmMLO02*, *GmMLO10*, *GmMLO18*, *GmMLO19*, *GmMLO20* and *GmMLO23 *genes. **Fig. S5.** Inheritance of induced mutations in *GmMLO02 *(A), *GmMLO19 *(B), *GmMLO20 *(C) and *GmMLO23 *(D) genes in T2 plants. Target sequences and PAMs are indicated in red and blue color, respectively. Inserted nucleotides are shown in yellow. Δ indicates targeted sequence changes: 0 for no change, - for deletion, + for insertion. **Fig. S6.** *E. diffusa *isolation and characterization. A Conidiophore and conidia of *E. diffusa *isolated from infected leaves. Scale bar = 10 μm. B Internal transcribed spacer (ITS) sequences of the collected *E. diffusa*. C Nucleotide BLAST result of the *E. diffusa *ITS sequence on GenBank, NCBI. **Fig. S7.** Powdery mildew resistant assessment of T2 offspring *Gmmlo *mutant lines under the net- house conditions. Infection levels were recorded using a 0 to 5 scale (described by Tran et al., 2015), which according to strong resistance to severe infection. Infection levels were calculated as the average of 20-30 biological replicates and three experiments. Statistical analysis was done using one-way ANOVA followed by a *post hoc *Turkey’s test. Significant difference was considered at *P *< 0.05. **Fig. S8.** Soybean seed production under the net-house conditions. A The total number of pods per plant. B The frequency of pods with 3 seeds. WT: Control line (DT26 cultivar); 3.1-3-41, 3.1-5-87 44, 15.1-2-2, 15.1-6-4: T2 offspring *Gmmlo *soybean mutant lines. Error bars indicate standard deviations, *n* = 9-14. Statistical analysis was done using one-way ANOVA followed by a *post hoc* Turkey’s test. Significant difference was considered at *P *< 0.05. **Fig. S9.** Transgene inheritance and segregation at different generations. A Representative results of herbicide leaf painting with glufosinate solution (200 mg/L) on wild-type (WT), herbicide resistant line (3.1) and herbicide susceptible line (3.1-3-41). B Gel electrophoresis of PCR amplicons of transgenes at T0, T1 and T2 generations. M: 1 kb DNA ladder; WT: non transgenic wild-type plant; (+): positive control (CRISPR/Cas9 vector); *bar*: herbicide resistance gene; 35S:pFGC: transgene region spanning pFGC vector and 35SPPDK promoter of pcoCas9. **Table S1.** Sequences of oligonucleotides and primer sets used in this study. **Table S2**. Genotypes of T0 mutant lines. **Table S3.** Inheritance and segregation of transgenes at different transgenic soybean generations.

## Data Availability

All the data generated or analyzed during this study are included in this published article and its supplementary information files. The ITS sequence of *E. diffusa* was deposited into GenBank with accession number OQ933656. The partial nucleotide sequences of *GmMLO* genes were deposited into GenBank with accession numbers OQ945362-OQ945367. The materials developed in this study are available from the corresponding author on reasonable request.
