# Supplementary material for: Enhancing powdery mildew resistance in soybean by targeted mutation of MLO genes using the CRISPR/Cas9 system
Source: BMC Plant Biol. 2023 Nov 3;23:533. doi: 10.1186/s12870-023-04549-5 (PMC10623788; doi:10.1186/s12870-023-04549-5)
Supplement: Supplementary file 1 — Additional file 1: Fig. S1. Transcriptomics analysis of GmMLO genes in different tissues of soybean plant. Data were obtained from RNA-Seq Atlas of Glycine max. Fig. S2. Induced mutation analysis of hairy roots. A Gel electrophoresis (agarose 1%) of GmMLO20 edited region in wild-type (WT) and hairy root samples (HR1, HR2, HR3) with large deletions. M: 1 kb DNA marker. Shifted bands in lines HR1, HR2 and HR3 indicated the induced mutations of targeted genes. B Sequencing result of the HR1 line for the edited regions of GmMLO20 compared to wild-type allele. Target sequences and PAMs are indicated in red and blue, respectively. Δ indicates targeted sequence changes: - for deletion. Clones indicate number of colonies with the respective alleles out of total of clones sequenced. Fig. S3. Soybean transformation procedure. A Cotyledons at 5 days on the co-cultivation medium. B, C Shoot induction at 14 and 28 days on the selection medium. D Shoot elongation. E Rooted plants on the rooting medium. F, G Leaf painting using 200 mg/L glufosinate. Fig. S4. Gel electrophoresis of PCR-amplicons of GmMLO target expanding regions at T1 generation. WT: Non transgenic wild-type plant; 3.1-2 to 3.1-5: T1 plants from 3.1 line; 15.1-1 to 15.1-6: T1 plants from 15.1 line; M: 1 kb DNA ladder. PCR products amplified by specific primers for extended regions of GmMLO02, GmMLO10, GmMLO18, GmMLO19, GmMLO20 and GmMLO23 genes. Fig. S5. Inheritance of induced mutations in GmMLO02 (A), GmMLO19 (B), GmMLO20 (C) and GmMLO23 (D) genes in T2 plants. Target sequences and PAMs are indicated in red and blue color, respectively. Inserted nucleotides are shown in yellow. Δ indicates targeted sequence changes: 0 for no change, - for deletion, + for insertion. Fig. S6. E. diffusa isolation and characterization. A Conidiophore and conidia of E. diffusa isolated from infected leaves. Scale bar = 10 μm. B Internal transcribed spacer (ITS) sequences of the collected E. diffusa. C Nucleotide BLAST result of the E. [file 12870_2023_4549_MOESM1_ESM.pdf]

Additional file 1

Enhancing powdery mildew resistance in soybean by targeted mutation of *MLO* genes using CRISPR/Cas9 system

Thao Phuong Bui[1,2], Huy Le[1,†], Dong Thi Ta[1], Cuong Xuan Nguyen[1,3], Ngoc Thu Le[1], Truong Thi Tran[4], Phuong Van Nguyen[2], Gary Stacey[3], Minviluz G. Stacey[3], Ngoc Bich Pham[1,5], Ha Hoang Chu[1,5] and Phat Tien Do[1,5*]

[1]Institute of Biotechnology, Vietnam Academy of Science and Technology, Hanoi, Vietnam

[2]University of Science and Technology of Hanoi, Vietnam Academy of Science and Technology, Hanoi, Vietnam

[3]Division of Plant Sciences, University of Missouri, Columbia, MO 65211, USA

[4]Legumes Research and Development Center, Vietnam Academy of Agriculture Science, Hanoi, Vietnam

[5]Graduate University of Science and Technology, Vietnam Academy of Science and Technology, Hanoi, Vietnam

[†]Current address: Department of Biology, Washington University in St. Louis, St. Louis, USA

* Correspondence: dtphat@ibt.ac.vn

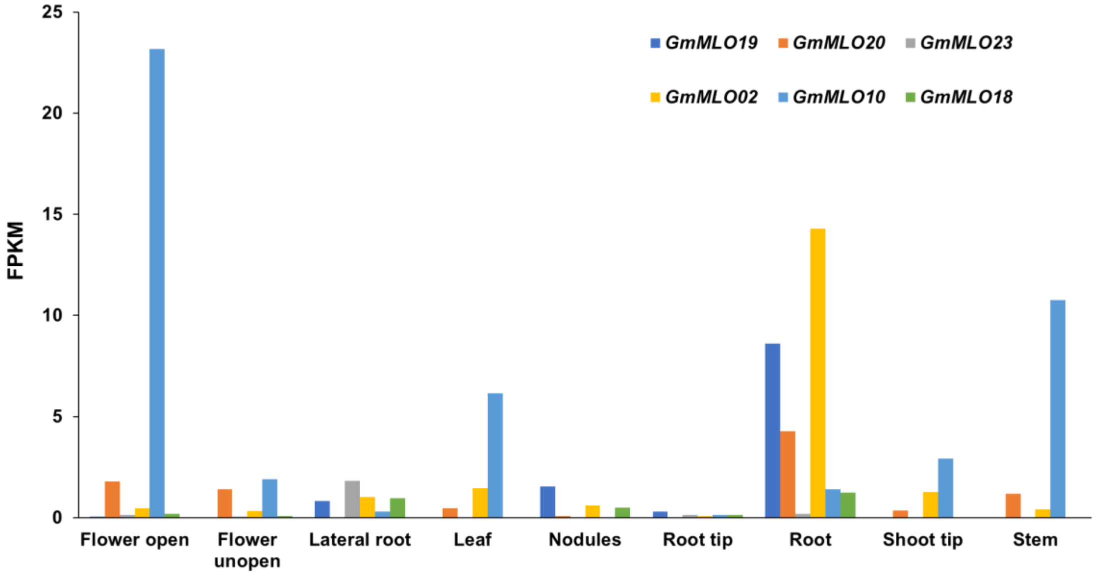

Fig. S1 Transcriptomics analysis of *GmMLO* genes in different tissues of soybean plant. Data were obtained from RNA-Seq Atlas of *Glycine max*

A

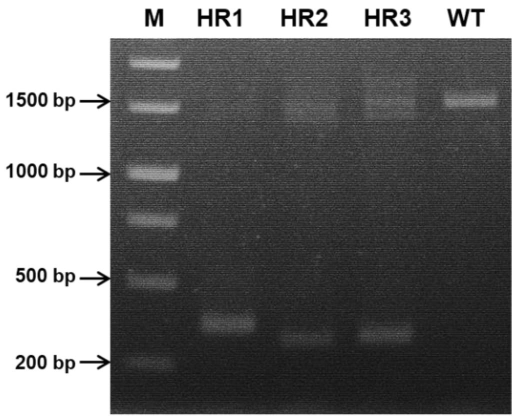

B

	Target 1		Target 2	Δ	Clones
GmMLO20 - WT	GATCCTGAGAGGTTCAGGTTTGCTAGGGATACA	1200bp	TCAAGGGTGCACCTGTGGTTGAGCCAGGAGATG		
HR1	GATCCTGAGAGGTTCAGG----------------	----------------------------	----------------------------CCAGGAGATG	-1241 bp	4/4

Fig. S2 Induced mutation analysis of hairy roots. **A** Gel electrophoresis (agarose 1%) of *GmMLO20* edited region in wild-type (WT) and hairy root samples (HR1, HR2, HR3) with large deletions. M: 1 kb DNA marker. Shifted bands in lines HR1, HR2 and HR3 indicated the induced mutations of targeted genes. **B** Sequencing result of the HR1 line for the edited regions of *GmMLO20* compared to wild-type allele. Target sequences and PAMs are indicated in red and blue, respectively. Δ indicates targeted sequence changes: - for deletion. Clones indicate number of colonies with the respective alleles out of total of clones sequenced

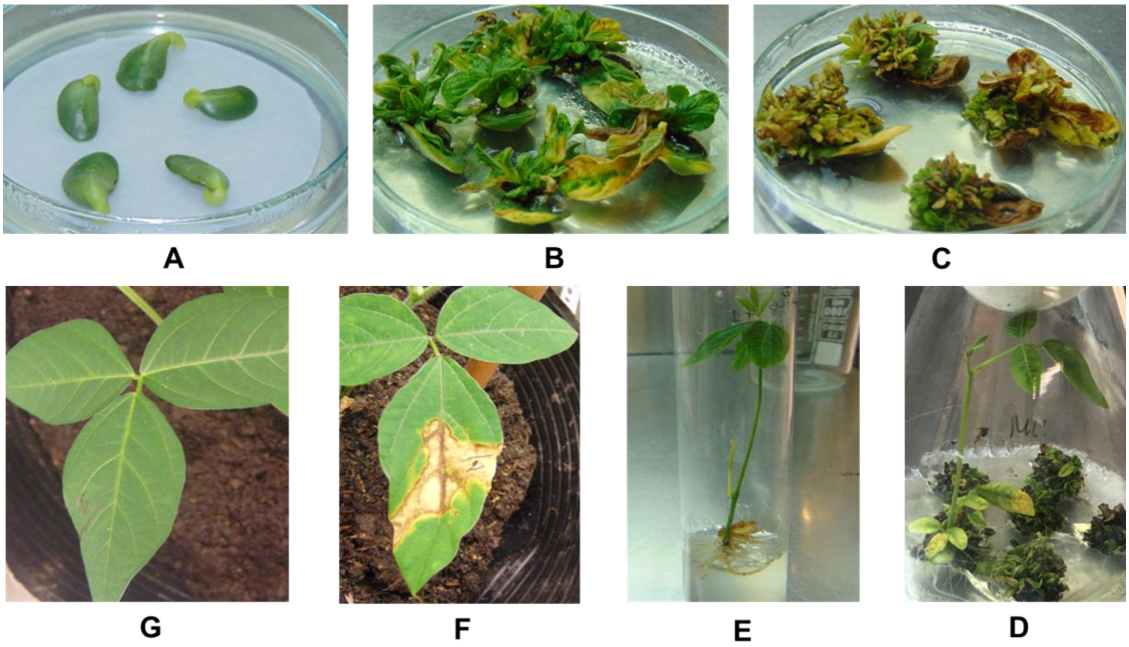

Fig. S3 Soybean transformation procedure. **A** Cotyledons at 5 days on the co-cultivation medium. **B, C** Shoot induction at 14 and 28 days on the selection medium. **D** Shoot elongation. **E** Rooted plants on the rooting medium. **F, G** Leaf painting using 200 mg/L glufosinate

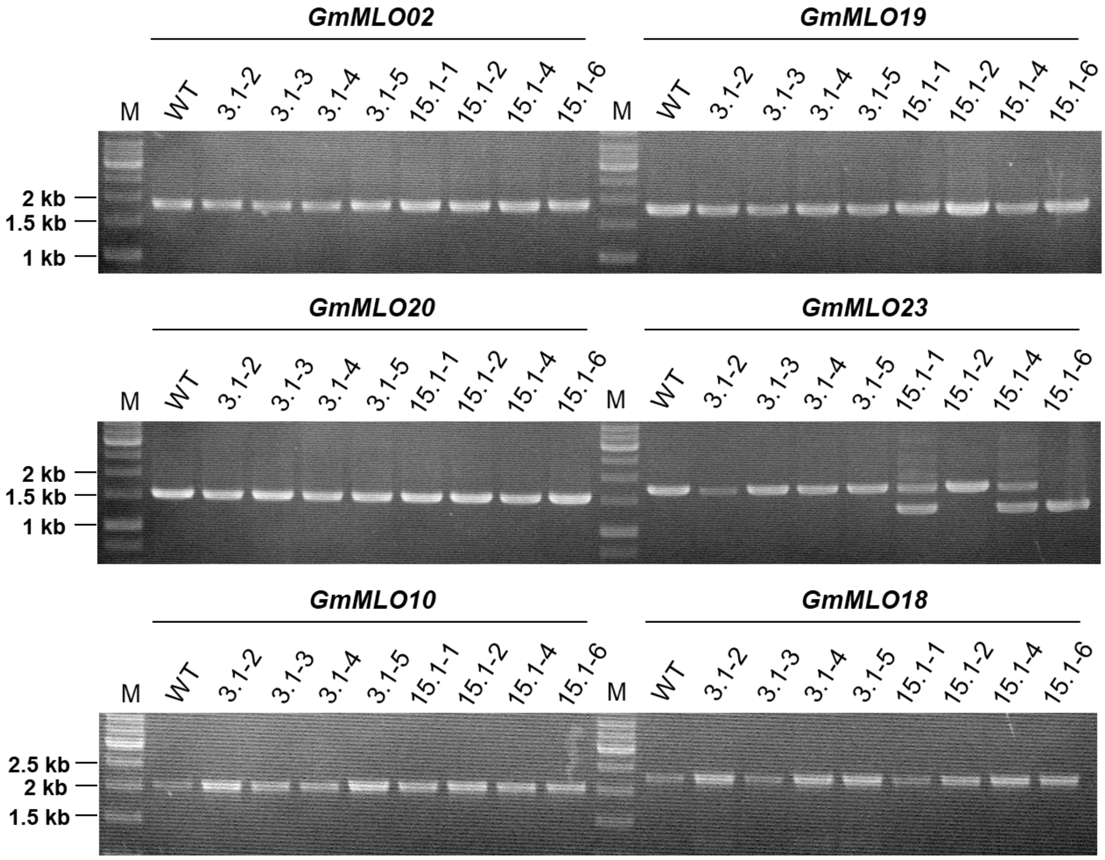

Fig. S4 Gel electrophoresis of PCR-amplicons of *GmMLO* target expanding regions at T1 generation. WT: Non transgenic wild-type plant; 3.1-2 to 3.1-5: T1 plants from 3.1 line; 15.1-1 to 15.1-6: T1 plants from 15.1 line; M: 1 kb DNA ladder. PCR products amplified by specific primers for extended regions of *GmMLO02*, *GmMLO10*, *GmMLO18*, *GmMLO19*, *GmMLO20* and *GmMLO23* genes

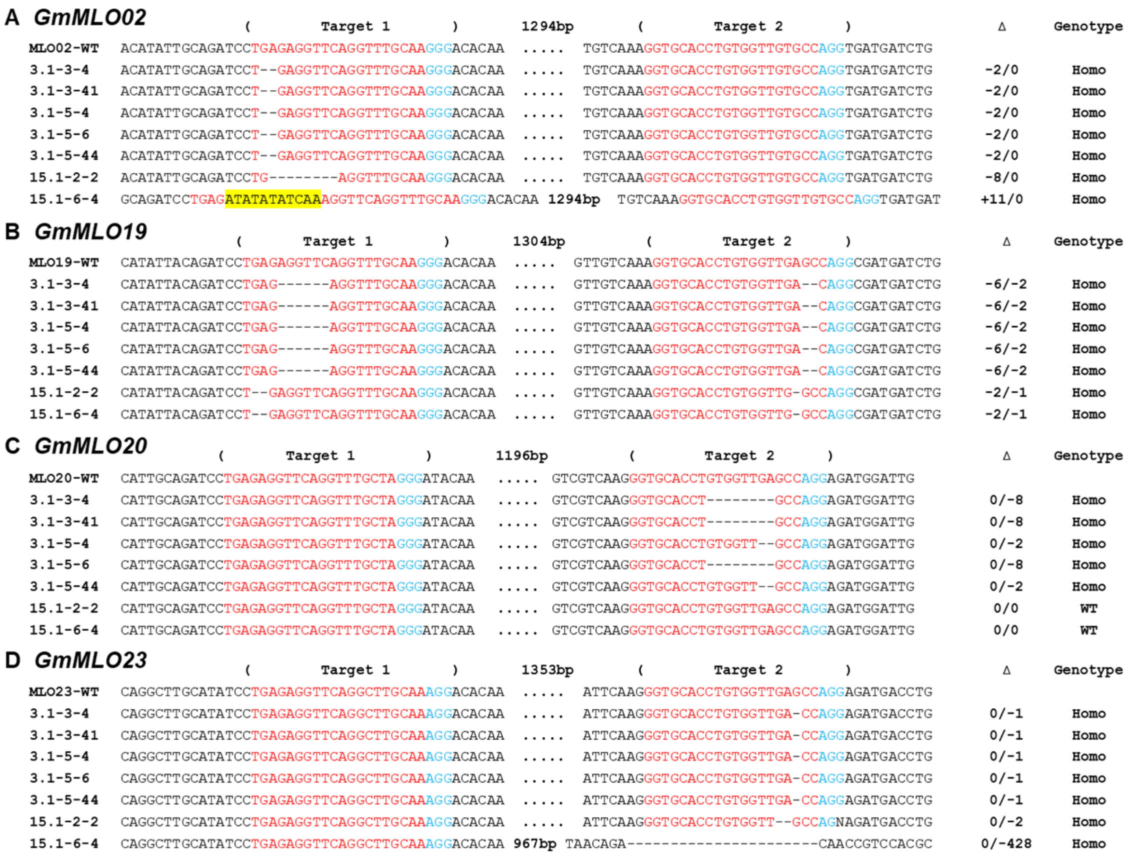

Fig. S5 Inheritance of induced mutations in *GmMLO02* (**A**), *GmMLO19* (**B**), *GmMLO20* (**C**) and *GmMLO23* (**D**) genes in T2 plants. Target sequences and PAMs are indicated in red and blue color, respectively. Inserted nucleotides are shown in yellow. Δ indicates targeted sequence changes: 0 for no change, - for deletion, + for insertion

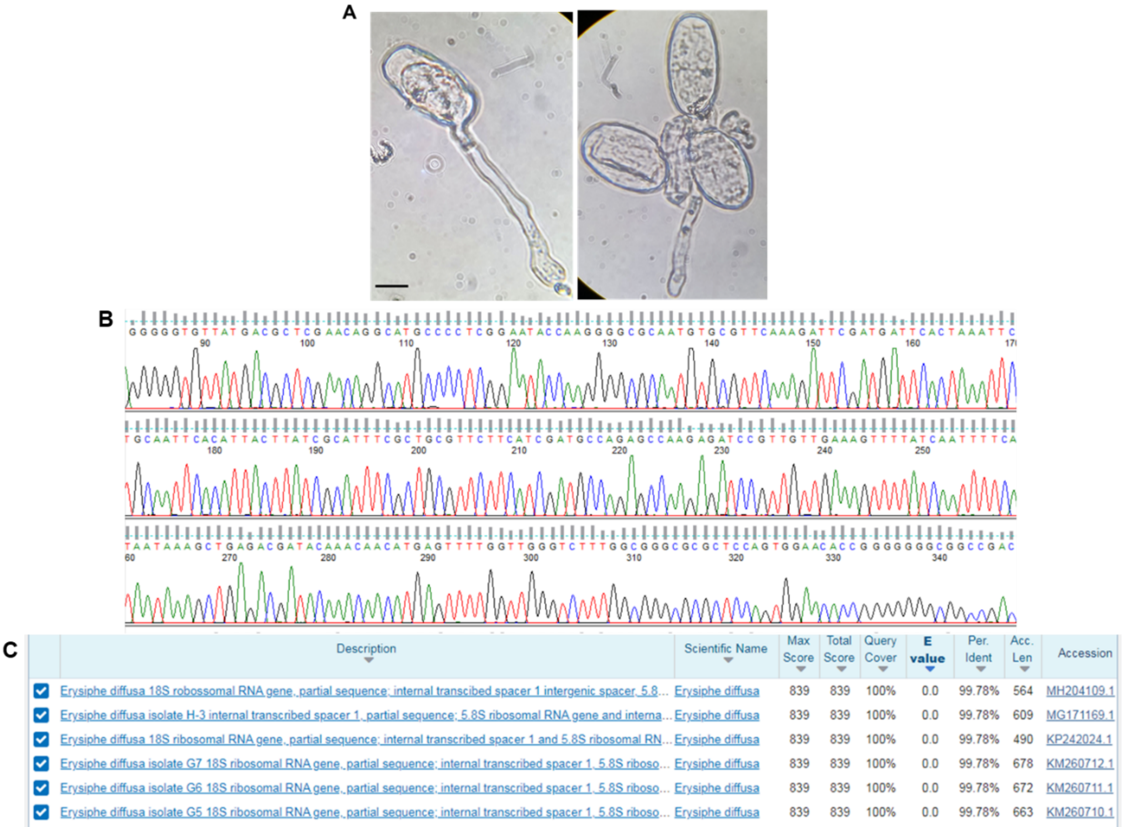

Fig. S6 *E. diffusa* isolation and characterization. **A** Conidiophore and conidia of *E. diffusa* isolated from infected leaves. Scale bar = 10 µm. **B** Internal transcribed spacer (ITS) sequences of the collected *E. diffusa*. **C** Nucleotide BLAST result of the *E. diffusa* ITS sequence on GenBank, NCBI

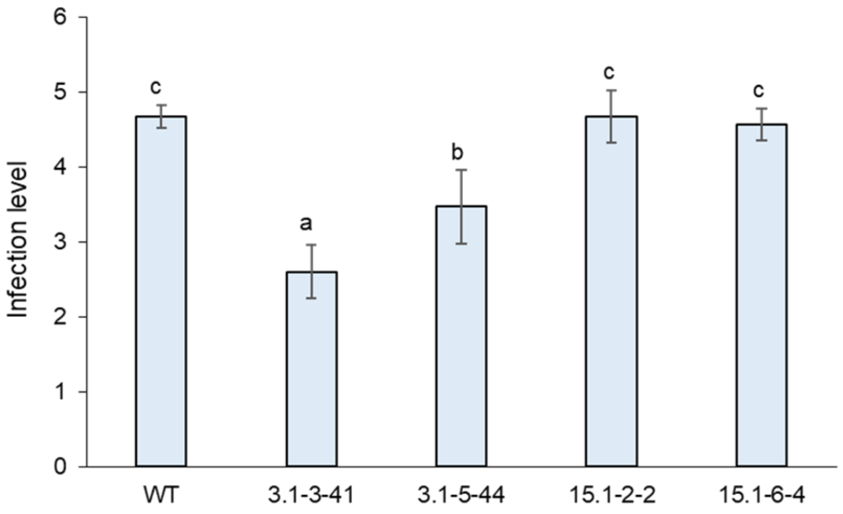

Fig. S7 Powdery mildew resistant assessment of T2 offspring *Gmmlo* mutant lines under the net-house conditions. Infection levels were recorded using a 0 to 5 scale (described by Tran et al., 2015), which according to strong resistance to severe infection. Infection levels were calculated as the average of 20-30 biological replicates and three experiments. Statistical analysis was done using one-way ANOVA followed by a *post hoc* Turkey's test. Significant difference was considered at $P < 0.05$

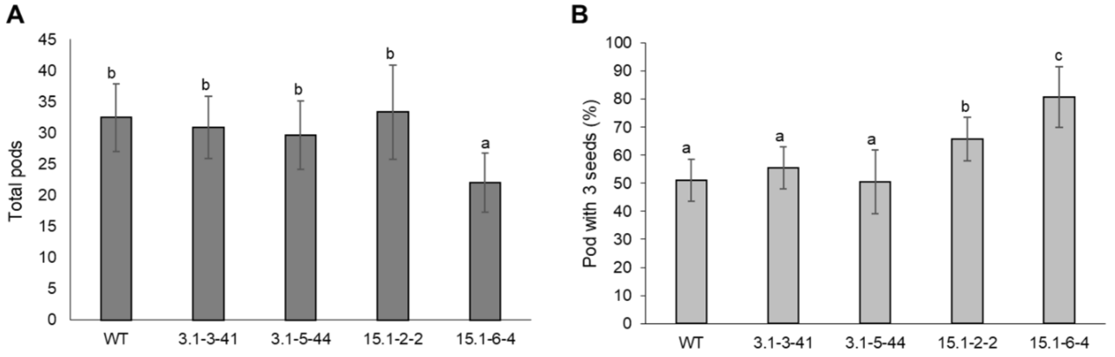

Fig. S8 Soybean seed production under the net-house conditions. **A** The total number of pods per plant. **B** The frequency of pods with 3 seeds. WT: Control line (DT26 cultivar); 3.1-3-41, 3.1-5-44, 15.1-2-2, 15.1-6-4: T2 offspring *Gmmlo* soybean mutant lines. Error bars indicate standard deviations, n = 9-14. Statistical analysis was done using one-way ANOVA followed by a *post hoc* Turkey's test. Significant difference was considered at $P < 0.05$

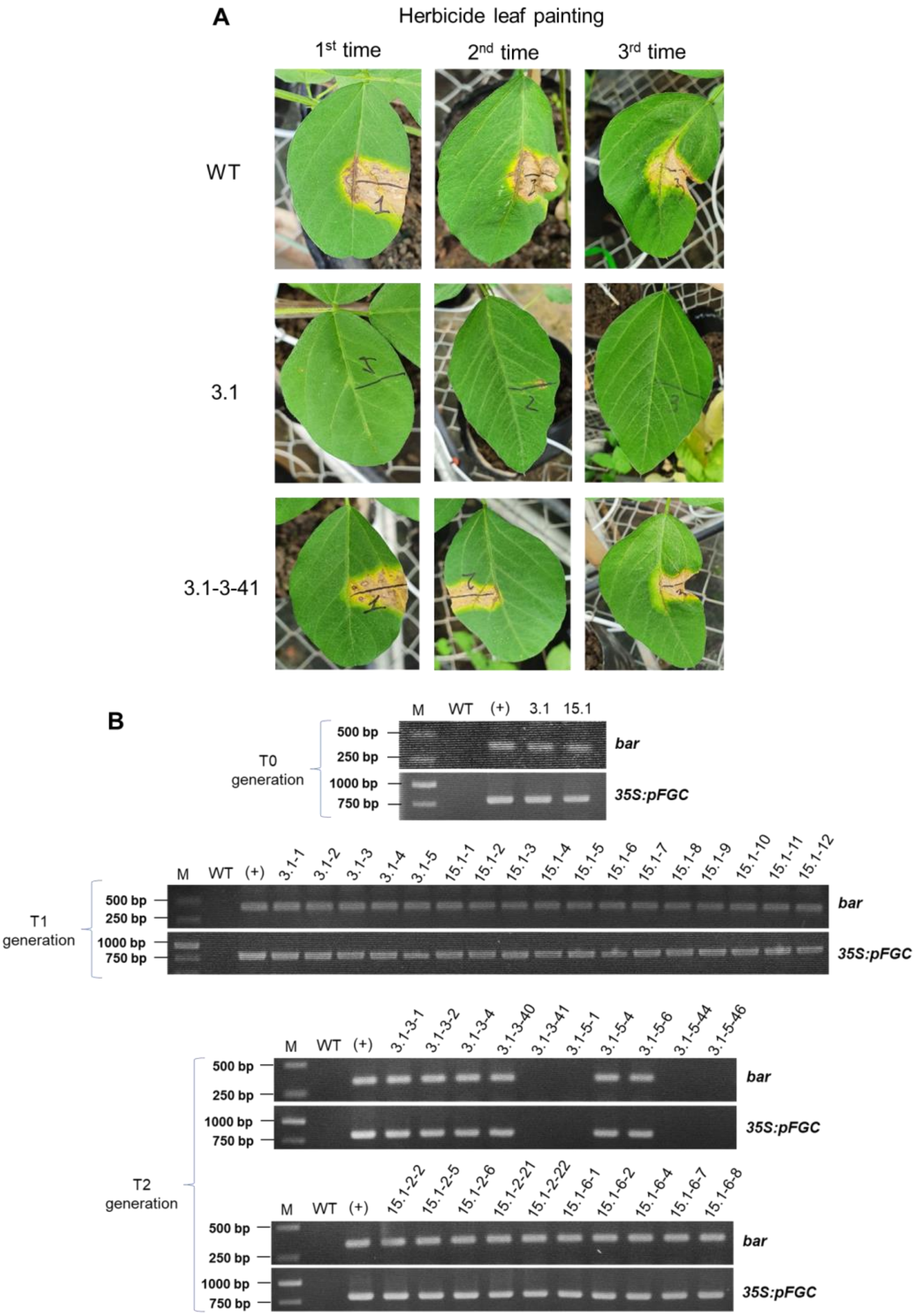

Fig. S9 Transgene inheritance and segregation at different generations. **A** Representative results of herbicide leaf painting with glufosinate solution (200 mg/L) on wild-type (WT), herbicide resistant line (3.1) and herbicide susceptible line (3.1-3-41). **B** Gel electrophoresis of PCR amplicons of transgenes at T0, T1 and T2 generations. M: 1 kb DNA ladder; WT: non transgenic

wild-type plant; (+): positive control (CRISPR/Cas9 vector); *bar*: herbicide resistance gene; 35S:pFGC: transgene region spanning pFGC vector and 35SPPDK promoter of pcoCas9.

Table S1 Sequences of oligonucleotides and primer sets used in this study

**Oligonucleotides and primers**	**5’-3’ sequence**	**Description**
MLO-gRNA1-F	**gttt**TGAGAGGTTCAGGTTTGCAA	Annealed to create gRNA for target 1
MLO-gRNA1-R	**aatc**TTGCAAACCTGAACCTCTCA	
MLO-gRNA2-F	**gttt**GGTGCACCTGTGGTTGAGCC	Annealed to create gRNA for target 2
MLO-gRNA2-R	**aatc**GGCTCAACCACAGGTGCACC	
bar-F	TACCATGAGCCCAGAACGACGCCC	Primer pair specific to *bar* gene.
bar-R	CTTCAGCAGGTGGGTGTAGAGCG	
35S:pFGC-F	TGTGCGTCATCCCTTACGTC	Primer pair specific to a transgene region spanning pFGC and 35SPPDK promoter.
35S:pFGC-R	GAAGGCGGGAAACGACAATC	
MLO-02-F	GCCATTGTTGGTTGGTTCCAT	Primer pair flanking the target region of *GmMLO02* (≈ 1905 bp)
MLO-02-R	TTGTTTCGTGGAAGCAGGAGT	
MLO-19-F	GGGTCTCTGTTTTTGCTTTCGGC	Primer pair flanking the target region of *GmMLO19* (≈ 1820 bp)
MLO-19-R	AGCAGAGAAACGACATGATTCCT	
MLO-20-F	TGTGTCATGCGTCTTGGATGT	Primer pair flanking the target region of *GmMLO20* (≈ 1555 bp)
MLO-20-R	GCATTCTGCAGCACAGAACC	
MLO-23-F	GTCCCATGCTTCTTCATGGTG	Primer pair flanking the target region of *GmMLO23* (≈ 1720 bp)
MLO-23-R	TGCTAATTGAAAGGCATTCTGC	
MLO-10-F	AGCATAGCCTTGTTAATTATGTTGA	Primer pair flanking the target region of *GmMLO10* (≈ 2077 bp)
MLO-10-R	ACTGTAACAAAAACAAAATGATGCG	
MLO-18-F	ACTAACTTGCCAGTGAAAACTACAT	Primer pair flanking the target region of *GmMLO18* (≈ 2317 bp)
MLO-18-R	TGCTAATTGAAAGGCATTCTGC	

Table S2 Genotypes of T0 mutant lines

Events	Gene					
	GmMLO02	*GmMLO10*	*GmMLO18*	*GmMLO19*	*GmMLO20*	*GmMLO23*
3.1	Homozygous	WT	WT	Chimeric	Chimeric	Chimeric
15.1	Biallelic	WT	WT	Homozygous	WT	Chimeric

Notes: Homozygous: genotype that includes two identical mutant alleles; Biallelic: genotype that includes two mutant alleles; Chimeric: genotype that includes one wild-type allele and more than one mutant allele

Table S3 Inheritance and segregation of transgenes at different transgenic soybean generations

Generation	Lines	Herbicide leaf painting	*bar*	35S:pFGC	Generation	Lines	Herbicide leaf painting	*bar*	35S:pFGC
T0	3.1	R	+	+	T2	3.1-3-1	R	+	+
	15.1	R	+	+		3.1-3-2	R	+	+
T1	3.1-1	R	+	+		3.1-3-4	R	+	+
	3.1-2	R	+	+		3.1-3-40	R	+	+
	3.1-3	R	+	+		3.1-3-41	S	-	-
	3.1-4	R	+	+		3.1-5-1	S	-	-
	3.1-5	R	+	+		3.1-5-4	R	+	+
	15.1-1	R	+	+		3.1-5-6	R	+	+
	15.1-2	R	+	+		3.1-5-44	S	-	-
	15.1-3	R	+	+		3.1-5-46	S	-	-
	15.1-4	R	+	+		15.1-2-2	R	+	+
	15.1-5	R	+	+		15.1-2-5	R	+	+
	15.1-6	R	+	+		15.1-2-6	R	+	+
	15.1-7	R	+	+		15.1-2-21	R	+	+
	15.1-8	R	+	+		15.1-2-22	R	+	+
	15.1-9	R	+	+		15.1-6-1	R	+	+
	15.1-10	R	+	+		15.1-6-2	R	+	+
	15.1-11	R	+	+		15.1-6-4	R	+	+
	15.1-12	R	+	+		15.1-6-7	R	+	+
						15.1-6-8	R	+	+

Notes: R: Herbicide resistant; S: Herbicide susceptible; (+): Positive for PCR-amplification with specific primers; (-): Negative for PCR-amplification with specific primers; bar: herbicide resistance gene; 35S:pFGC:transgene region spanning pFGC vector and 35SPPDK promoter of pcoCas9
